# Pre-Harvest Modelling and Mitigation of Aflatoxins in Maize in a Changing Climatic Environment—A Review

**DOI:** 10.3390/toxins12120768

**Published:** 2020-12-04

**Authors:** Tamás Dövényi-Nagy, Csaba Rácz, Krisztina Molnár, Károly Bakó, Zsombor Szláma, Ákos Jóźwiak, Zsuzsa Farkas, István Pócsi, Attila Csaba Dobos

**Affiliations:** 1 Agrometeorological and Agroecological Monitoring Centre, AKIT-DTTI, University of Debrecen, H4032 Debrecen, Hungary; raczcs@agr.unideb.hu (C.R.); molnark@agr.unideb.hu (K.M.); bakok@agr.unideb.hu (K.B.); szlama@agr.unideb.hu (Z.S.); dobosa@agr.unideb.hu (A.C.D.); 2 Digital Food Institute, University of Veterinary Medicine Budapest, H1078 Budapest, Hungary; jozwiak.akos@univet.hu (Á.J.); farkas.zsuzsa@univet.hu (Z.F.); 3 Department of Molecular Biotechnology and Microbiology, Institute of Biotechnology, Faculty of Science and Technology, University of Debrecen, H4032 Debrecen, Hungary; pocsi.istvan@science.unideb.hu

**Keywords:** aflatoxin contamination, maize, climate change, field modelling, agro-technical measures, temperate climatic zone

## Abstract

Aflatoxins (AFs) are harmful secondary metabolites produced by various moulds, among which *Aspergillus flavus* is the major AF-producer fungus. These mycotoxins have carcinogenic or acute toxigenic effects on both humans and food producing animals and, therefore, the health risks and also the potential economic damages mounted by them have led to legal restrictions, and several countries have set maximum allowable limits for AF contaminations in food and feed. While colonization of food and feed and AF production by *A. flavus* are highly supported by the climatic conditions in tropical and subtropical geographic regions, countries in the temperate climate zones are also increasingly exposed to AF-derived health risks due to climate change. In the present study, we have reviewed the available mathematical models as risk assessment tools to predict the possibility of *A. flavus* infection and levels of AF contaminations in maize in a changing climatic environment. After highlighting the benefits and possible future improvements of these models, we summarize the current agricultural practices used to prevent or, at least, mitigate the deleterious consequences of AF contaminations

## 1. Introduction

Aflatoxins (AFs) are dangerous secondary metabolites produced by various *Aspergillus* species, spoiling various crops, representing serious health threats to both humans and domestic animals and causing considerable economic losses worldwide. AFs are produced predominantly by *A. flavus*, *A. parasiticus*, *A. nominus* and some other moulds as well, with *A. flavus* being the main AF producer [1]. This fungus may infect crops including e.g., peanut, cottonseed, maize and various other nuts, cereals, spices, and dried fruits. While *A. flavus* has both toxigenic and non-toxigenic strains, under specific environmental conditions it may produce different AFs, with aflatoxin B1 (AFB1) being the most potent natural carcinogen known [2]. Apart from being highly carcinogenic, AFs can also be acutely toxic or even fatal for both livestock and humans if ingested in sufficient amounts.

Contaminations are more frequent and more severe in tropical and subtropical areas where temperature and humidity levels support growth of and infection by the fungus, as well as its AF production. However, under favourable climatic conditions in temperate climatic zones, especially during hot, dry periods, crops grown in those areas are also increasingly exposed to the risk of AF contamination.

Public health considerations have led to legal restrictions and several countries have regulations in place to define maximum permitted AF and AFB1 levels in food and feed. (For example, maximum 0.1–12 μg kg^−1^ AFB1 in the EU, 20 ppb total AF in the USA or 5–20 μg kg^−1^ AFB1 in China [3,4,5].) Exceeding these acceptable limits may result in economic losses due to limited trade and economic opportunities: e.g., AFs are a major reason for rejecting imports of various products in the EU [6]. This has driven the development of various preventive measures, predicting tools and mitigation technologies (see Section 3). Preferred prevention methods include good agricultural practices (see Section 4), proper storage conditions and manufacturing protocols.

In order to assist policy makers and growers, several risk assessment tools have been developed for various crops. There are risk maps based on historical data, as well as empirical and mechanistic models available to predict AF levels and risk of exceeding regulatory thresholds. The peanut was the model developers’ first main focus of interest because it is a main staple in African countries where some of the most severe cases occurred. However, in many European countries, maize (*Zea mays* L.) is a much more important crop and *A. flavus* and AF models for maize as a crop of interest are also available.

Development of mechanistic models requires good understanding of the pathogen’s life cycle. *A. flavus* can colonize various plant species and affect seeds above and below ground level, resulting in diverse host-parasite interactions. The fungus generally survives in the soil or on plant residues. The major inoculum source to start the infection cycle is overwintering sclerotia. Flowering is the stage when maize as the host plant becomes susceptible to *A. flavus* colonization and infection occurs by air-borne conidia through the silk [7]. Colonization of maize kernels may continue throughout the growing season and is supported by insect damage [8]. Drought stress for the host plant under high temperatures and low humidity conditions, are associated with high aflatoxin contamination. *A. flavus*, in such an environment, may become the dominant pathogen in maize [1,9,10].

The objective of the present study was to review the scientific literature regarding (a) the relation of climate change and *A. flavus* growth, infection and AF contamination in the Pannonian and the corresponding North-Mediterranean agro-ecological zone, (b) the possible solutions for predicting the level of AF contamination in maize in a changing climatic environment. The study especially focused on mathematical models. Finally, we summarize (c) the agricultural practices available to prevent and mitigate pre-harvest AF contamination when the models indicate an elevated risk.

## 2. Environmental and Ecological Background

### 2.1. Pathogen Occurrence

It is clearly seen that the incidence of aflatoxin-related health crises varies from year to year and is largely dependent on climatic factors [10,11,12,13]. Countries in the temperate climatic zone that today appear to be safe may therefore, as well, become more vulnerable to risk of disease and/or loss in crop production due to contamination, as climatic conditions change [14,15].

In the past, serious aflatoxin contamination was detected particularly in the developing countries of the tropical climatic zone. Benkerroum [11] reported past major outbreaks of aflatoxicosis associated with maize-based foods mainly, which occurred in West-India (1974), Kenya, 1981 and 2004, and Tanzania (2016, 2017) [12,13]. Nevertheless, the presence of the pathogen is not limited to the tropical region.

*A. flavus* has been proved to be present in most soils in Northern Italy since 2003 [16] (even in fields without AF issues in recent years), and the authors devised the AFLA-maize model with this in mind [17]. In a study by Leggieri et al. [18], the incidence of AFs exceeded 75% across all samples in Northern Italy over the years 2009–2011.

Since 1994, presence of the pathogen is unquestionable in Croatia, first detected from flour samples [19]. By 2013, the percentage of contaminated maize samples has already exceeded 38.1%, and in almost one third of the cases, mycotoxin levels proved to be higher than the maximum permitted level [20].

In Serbia, *A. flavus* and AFB1 were isolated from 18.7% and 18.3% of the samples, respectively, [21]. Investigation of the influence of hotter and drier weather conditions on levels of aflatoxin in maize in 2015 found as high as one third of the samples unsuitable for human consumption due to overall AF concentration higher than 10.0 μg kg^−1^ [22].

Data published in the most recent decade [23,24,25,26,27,28] confirmed the presence of toxigenic strains of *A. flavus* and aflatoxin contamination in agricultural products and soil samples in Hungary and other neighbouring countries of the Pannonian agro-ecological zone.

### 2.2. The Role of Ecophysiological Factors in Aflatoxin Contamination

Germination, growth, infection and toxin production by *Aspergillus* species are determined by relatively few measurable parameters. In laboratory experiments, the variables taken into account are temperature, moisture content (water activity of the kernel: a_w_, and/or relative humidity), CO_2_ concentration and pH. It is important to note that in vitro experiments may substantially differ from field conditions where the host plant and pathogen culture are equally exposed to further, often more complex ecological factors.

#### 2.2.1. Laboratory (In Vitro) Findings

According to Medina et al., the main driving force of AF production is a combination of three environmental factors, i.e., ambient temperature, water activity and elevated concentration of CO_2_ [29]. While three-way interactions between CO_2_ concentration, temperature and water activity do not have a significant effect on the growth of *A. flavus*, they do stimulate the biosynthesis of phenotypic AFB1 [30]. Detailed in vitro data have been published concerning the optimal range of temperature and a_w_ for germination, growth and toxin production: *A. flavus* germinates under a wider range of temperature and water activity, while growth and toxin production have a narrower tolerance with an optimum of 35 °C/0.95 _aw_, and 33 °C/0.99 a_w_, respectively [31]. As a synopsis of further collections of data, Table 1 summarizes the range of ecological needs of growth and toxin production by *A. flavus*.

It is highly likely that ambient physiological stressors stimulate the mycotoxin production of various fungal species [34,35]. Two peaks can be identified in mycotoxin biosynthesis in relation to a combination of environmental parameters (temperature × a_w_ regimes). In a two-factorial experiment, microarray analysis showed that toxin production by *Penicillium verrucosum*, *Fusarium culmorum* and *Aspergillus parasiticus* reaches one major peak under the optimal growth conditions, and a second, lower one under mild stress conditions [34]. Findings were later confirmed by another study [36], suggesting a relative dominance of aflatoxin G1 production by *A. parasiticus* between 17–30 °C and marking water activity as a driving environmental parameter. In the case of AFB1, the key parameter was temperature. Ramirez et al. [37] and Giorni et al. [38] drew attention to the importance of different forms of water stress, of which matric stress is more likely to stimulate toxin producing fungi than solute stress.

#### 2.2.2. Effect of Climatic Conditions in Field Environment

Field studies agree on the predominant role of drought and high temperatures in elevated aflatoxin production in maize [18,20,22]. Despite their importance in providing valuable information on how theories work out in the complexity of ecophysiological factors and agronomic practice, these studies are still limited in number [39,40,41] compared to in vitro experiments (for further references see Section 4).

An analysis of 3000 maize samples harvested in Serbia during 2012–2016 [14] revealed that elevated AF concentration was pre-eminently a consequence of climatic extremes, such as serious drought and hot summer temperature. As an indirect effect, growth of maize plants suffers a setback due to heat and drought stress. This facilitates the infection of maize plants by any pathogenic fungi while hot and dry atmospheric conditions proved to be utterly favourable for the growth of *A. flavus* [42,43,44,45,46,47,48,49,50,51,52,53,54]. Dispersal of *A. flavus* and the infection itself requires airborne spores in the ambience of maize ears [55]. Leaf and soil wetness have a negative influence on the magnitude of infection, as well as falling precipitation that washes conidia and spores out of the atmosphere [55,56]. Since dry plant and soil surface are mostly associated with high to hot air temperature the latter is generally reported as a root cause of serious outbreaks of infection [45,46,57].

Some mycotoxigenic species may be able to better adapt to stress than others and, therefore, this feature fundamentally determines ecological dominance in any given environment. Climatic extremes, such as drought and hot temperature, may not have a negative effect on the growth and toxin production by xerophilic fungi [58,59]. It was also found that the diversity of *Aspergillus* strains is strongly linked to the amount of rainfall and, accordingly, soil wetness and soil temperature. Consequently, AF production may also alter periodically and significantly at a given location [60,61,62,63,64]. High AF contamination in maize is often associated with weather conditions, as the preceding period was extremely warm, dry, and was characterised by a very low average rainfall. All of the aforementioned favour mould formation and AFB1 presence in maize. In accordance with the findings of preceding studies worldwide, hot and dry weather patterns with drought episodes proved to be typically favourable for higher than average AFB1 contamination in maize production [20].

### 2.3. Effects of Climate Change on Crop Production-Yields

Impact of climate change on agricultural productivity is detailed in several resources [65,66]. Agricultural production of the tropical and subtropical maize producing countries is generally expected to decrease parallel to a +3 °C global warming scenario, whereas it may result in a considerably higher production potential in the temperate climate zones [67]. Predictions suggest drier and hotter summers in Western and Atlantic Europe, as well as in the Mediterranean region, with at least the risk of decrease in crop production. In parts of Asia, South America and the US, climatic conditions may be more beneficial to crop yields, similarly to Northern and Central Europe [68]. Yield responses in different emission scenarios have been estimated by Iglesias et al. [69] for each agroclimatic zone of Europe by the end of the present century. An 11–33% increase was projected for the Southern continental zone (Hungary, Moldova, Romania, Serbia, Slovakia, Ukraine), and a similarly positive change in the yield of wheat, maize, and soybean crops for the Boreal, Alpine and parts of the North-Central Atlantic zones.

Although findings highly correspond with later reports in terms of growing potentials, the critical role of increasing yield variability is as well worth attention. More complex environmental parameters, such as fluctuations of groundwater level, are rarely taken into account in large scale predictions despite their effect on yield potential having already been proved. In Hungary, maize yield variability was found to be 18–38% dependent on ground water decrease between 1986 and 2010 [70]. Added to the effect of higher temperatures, it has already slowed down crop production during the past decades [71]. By means of statistical tests, Moore and Lobell [72] introduced a novel method to substantiate that temperature and precipitation trends related to climate change caused a decrease in barley and wheat yields from the late 1980s, and a slight increase in that of sugar beet and maize. The question raised by the altering climatic conditions is how much an uncertain increase in yield would be able to compensate losses due to toxin contamination in the years of outbreaks.

### 2.4. Effects of Climate Change on Crop Production—A Risk Factor in Food Safety

A review presented by Paterson and Lima [73] acknowledges the possibility of plant pathogen fungi, such as *A. flavus*, spreading towards currently colder than optimal locations within the temperate climate zone. Colder climates may reach optimal temperatures for *A. flavus* in most global warming scenarios, unlike very cold, Nordic countries. In already hot climates, more frequent drought may result in higher mycotoxin production. In continuation of their previous review, Paterson and Lima [74] confirm research priorities already stated by Magan et al. [75], highlighting the importance of monitoring the geographical spread of mycotoxigenic fungi and maintaining quality databases prior to model development and further analysis of the effect of environmental factors. In addition to the inevitable value of in vitro data, there is an urgent need for field experiments to provide reliable in vivo data sources, as well.

The estimated decrease in crop yield due to pathogens may reach 16%, with possible further specific losses in quality on account of the elevated mycotoxin contamination as a result of extreme maximum temperatures and frequent droughts [76]. Based on local reports from 1960, Bebber et al. [77] depict a 2.7 km yearly shift towards the poles in the habitats of crop pests, including plant pathogen fungi. Losses in crop production may surge in larger regions extended to the higher latitudes, mainly due to rapid global warming. In a comprehensive study on global mycotoxin risks versus climatic and other environmental parameters, Wu et al. [78] hypothesize a possible increase in aflatoxin and fumonisin contamination levels in maize for the US, predicting a similar process for the European maize producing regions.

Regarding air pollution as a further concern accompanying present time atmospheric changes, elevated CO_2_ level and SO_2_ pollution very likely influence host plant-pathogen relations in virtue of acidity. Acid environments may also have an effect on the growth of phylloplane fungi, either caused by sulphur dioxide or carbon dioxide [79,80,81].

For European maize and wheat production, the increasing degree of aflatoxin contamination has been predicted to be promoted as a major issue in food safety. A prediction model conducted by Battilani et al. [82] draws attention to a possible significant increase in ARI (aflatoxin risk index) in both a +2 and +5 °C climate change scenario for the next 100 years, especially for Eastern Europe, the Balkan Peninsula and the Mediterranean region. Chauhan et al. [83] addresses the interactions between environmental conditions and maize plants. For the quantification of climatic risk in different locations of Australia, agronomic practices that minimise the effects of drought and high temperature were also taken into account and found to reduce ARI. It is also recommended for the processing industry to keep the produce of higher and lower risk areas separate, either in field or regional scale. A number of food safety issues may arise in staple foods from mycotoxin contamination to pesticide residues. As a response, quality monitoring, strict policies and regulation in the food chain will be of critical importance in the near future, urging adaptation and the establishment of a “climate-smart food system” [84,85,86,87].

## 3. Risk Assessment and Modelling

### 3.1. Role and Significance of Risk Assessment and Modelling

The role of risk assessment and modelling is to identify the risk factors and levels associated with specific growing seasons on the spread of *A. flavus* and AF contamination. With the help of these tools, prevention and mitigation of economic losses, animal and human health issues are possible.

Surveying AF is relatively slow and expensive, and since AF levels are usually determined after harvest, the results are not available for implementing within the season for pre-harvest mitigation [88]. Nevertheless, risk assessment tools may help to identify high and low risk areas and growing seasons, the identification of which is essential to take preventive measures or to increase the number of samples analysed for AF concentration.

The types of risk assessment tools available range from risk maps based on simple historic statistical data to sophisticated mechanistic models simulating the life cycle of *A. flavus*. However, with climate change (e.g., air temperature levels and rainfall distribution changing), statistical risk assessment solutions based on historical data may no longer identify risks as expected. Similar to empirical models, which may need to be recalibrated. Mechanistic models, on the other hand, are able to incorporate these changes.

### 3.2. Modelling

Modelling of interactions between environment and host plant during the growing season can enable assessment and quantification of pre-harvest AF risk and its potential management [83]. While ‘mycotoxin’ is a hot topic, efforts to model the risk of contamination in crops are still limited. Battilani [89] found that only 10% of mycotoxin related articles mention the keyword ‘model’ and only 1% of them mention ‘crop’, indicating that most models simulate AF production in vitro, without a target crop. Additionally, since issues associated with AF are more common in tropical and subtropical regions and affect staple crops in those areas, model developers’ main focus was understandably peanut and pistachios. These crops are regularly contaminated as opposed to crops commonly grown in areas with temperate climate. Thus, if one is looking for a specific fungus on a specific crop, the number of publications available is very limited. Battilani et al. [17] found only two different approaches regarding modelling of *A. flavus* on maize in the field.

There can be several reasons behind this: difficulties of model development, the low interest in modelling, the unclear view on their support and the limited trust in their benefit.

Nevertheless, modelling the interactions between *A. flavus*, e.g., maize as the host plant and the environment during the growing season could enable prediction of the risk of AF contamination until harvest. This information is crucial for optimizing crop management both before and after harvest, leading to a mitigation of economic losses and consumer exposure.

#### 3.2.1. Types of Models

Mathematical models can be classified into categories based on e.g., their iteration frequency and inner working mechanism. Crop modelling, as well as the AF models introduced in this paper, mostly involve discrete modelling, which means that events occur and variables change at some discrete points of time. There are daily, weekly or decadal models, but more frequent iteration is also possible if the input variables (e.g., hourly weather parameters) are available.

Empirical models are developed by statistically analysing data collected in field trials and are used to describe how the driving factors (e.g., air temperature, rain) and the response variables (e.g., AF levels) are linked. Such a model will characterize the specific area, climate and seasons involved in the model development and may need recalibration when used in a different environment.

Mechanistic models try to enumerate the cause-and-effect relationships of the environmental variables and different states of a system. This way, they can describe and explain biological processes [90].

Model development usually involves both these approaches: in practice, mechanistic models are usually validated against field data using the principles of empirical models [91].

#### 3.2.2. In Vitro vs. In Vivo Fungal Growth Modelling

Modelling of fungi has followed that of bacteria with some lag due to difficulties in model construction [92]. Since bacteria reproduce by fission, with growth normally taking place only on surfaces or homogeneously through a liquid medium, modelling is relatively easy. However, fungal growth involves germination, hyphal extension and penetration of the physical three-dimensional matrix of parts of the host plant or medium [93], making the model building task more difficult.

Despite the difficulties, mathematical modelling has been used to predict the extent of fungal growth and invasion as being dependent on environmental conditions [94,95,96,97,98,99,100,101,102,103]. Unlike strictly empirical models which are limited to the conditions in which an experiment is performed, semi-mechanistic and mechanistic models can provide insight into the behaviour of a biological system in more natural and uncontrolled environments [95].

A complex mathematical model that integrates the effects of temperature, water activity, pH and colony size on mould growth and aflatoxin production under laboratory conditions was proposed [95]. Various other authors have developed probability, empirical and mechanistic/semi-mechanistic models for a variety of toxigenic and spoilage fungi [92,96,97], as well as specifically for *A. flavus* and AF prediction [90,98,99,100,101,102,103]. These models are usually based on in vitro trials and interactions between the fungus and input variables like temperature, pH and water activity.

While some of the models are able to simulate aflatoxin contamination well under in vitro conditions, in most cases on artificial culture media, their application under field conditions and on living plant parts needs further evaluation. Magan et al. [75] underlines the importance of the validation of existing predictive models. Model tests should include the evaluation of recent changes in the main driving climatic parameters, namely temperature, CO_2_ concentration and a_w_, and their interactions, as well.

Probst and Cotty [104] pointed out the lack of correlation between the results of in vitro and in vivo experiments, cautioned on their use for predicting contamination in maize grains, and suggested crops of direct concern as culture media (e.g., maize grain).

Battilani et al. [105] found a discrepancy in the optimal a_w_ range of in vitro vs. in vivo AF production. While in vitro results on artificial media suggest that AF levels decrease when a_w_ decreases, field data show an incrementing AF production at kernel humidity below 28% [1].

An important task to potentially improve AF models is to resolve this discrepancy between in vitro and in vivo results. However, the very nature of field trials, where factors, such as, e.g., weather or pests cannot be controlled in such a way as done in vitro, render the acquisition of proper field data difficult.

#### 3.2.3. Aflatoxin Prediction in Different Crops

One method of risk assessment is to apply geostatistical methods to develop a predictive tool for identifying problematic regions, with a longer-term view. A sensitivity analysis carried out by Kerry et al. [106] suggests that at least 20 years of data are desirable to generate reliable space–time summaries of AF risk. Although these tools based on historic data can be useful to identify areas with high risk of AF contamination, they are usually unfit to predict AF levels in a changing climatic environment.

Similarly, empirical models or simple empirical equations which use meteorological data like temperature (daily mean or min–max) and precipitation, are mostly only valid to the area and crop on which the research was conducted.

Mechanistic models, on the other hand, try to represent complex natural processes through the underlying relations of the parts of the system and their numeric representation. Thus, to develop a mechanistic *A. flavus*/AF model, the knowledge and enumeration of the life cycle of *A. flavus* and the factors of AF production are necessary.

Although this paper’s main focus is maize, it is worth mentioning some models targeting other crops. Since *A. flavus* and AFs are more prevalent in areas with a tropical climate, a large number of model developments concentrate on peanuts. While peanut, as an important crop in the southern USA and as a main staple in Africa, might seem less important in other countries in temperate regions, investigating the experiences of at least some peanut models can be useful for models with maize in focus [107,108,109,110,111,112,113,114]. Furthermore, some peanut models are direct predecessors of maize models, as they were used as a starting point by maize model developers [83,109,114].

#### 3.2.4. AF Models in Maize

Multiple mechanistic models were developed to predict pre-harvest AF contamination in field grown maize, based on the climatic dependencies of the *A. flavus* to colonize maize and produce AFs (Table 2) [10,17,83,115,116,117].

Model 1 (Chauhan et al., 2008): A simulative model, using principles previously used for peanuts, was developed to quantify climatic risks of AF contamination in maize as an extension of the APSIM modelling environment [83,114]. The original model used as a starting point to develop this model was the peanut model by Wright et al. [109]. The calculation of the model’s output, an aflatoxin risk index (ARI), is based on daily climatic and soil data during the maize grain filling period: air temperature, radiation, rainfall, soil water and soil nitrogen to simulate maize growth and yield. The fact that the model does not utilize water activity as a driver for aflatoxin production (in contrast to in vitro models) is a major benefit for farmers since a_w_ is not a commonly used parameter in agriculture and is hard to assess on a daily basis. The simulation identifies the coincidences of <20% available soil water and air temperature between ≥22 and ≤35 °C from the start of the grain filling stage, which are accumulated in the aflatoxin risk index. The risk computed by the model was found to be related to the level of aflatoxin contamination, but was evaluated only for a limited number of locations. The model assumes inoculum always being present in the soil and sporulation, dispersal and germination non-limiting, which is a reasonable assumption given that the temperature range favourable for the growth of maize also favours germination and growth of *A. flavus* [118]. However, this model completely ignores the life cycle of *A. flavus* and can be considered mechanistic only on the basis of the maize phenology part. Thanks to the modelling framework, differences, e.g., between hybrids, plant density, and sowing dates can be expressed, but not the severity of insect damage. Additionally, the temperature and soil water thresholds used were derived from in vitro studies with peanuts as a substrate. Therefore, verification of these values for maize may increase the accuracy of the model. This work has been done in a revision of this model [115].

Model 2 (Battilani et al., 2008): Battilani et al. [10] used an aridity index (AI) to estimate the probability of AF contamination of the harvested maize above the European legal limit. This empirical approach, requiring easily available data as input, could give a rough prediction. This predictive system computed an AI to summarise meteorological conditions which was used to estimate the probability of AFB1 contamination. The model’s input variables were daily mean air temperature and rain, and these were aggregated in ten-day periods to calculate the AI. The temperature and rain data were organized as decadal climatograms and the AI was computed as the difference of area under rain curve and area under temperature curve. Values of AI lower than zero indicate aridity. A logistic regression, using the decadal AI as independent variable, gave warning on AF contamination with 64% correct predictions. This rough predicting tool uses easily available weather data, is easy to calculate, but as an empirical model may have limited value in other areas. Without a mechanistic approach on part of the host plant and/or *A. flavus*, this model does not incorporate, e.g., the differences in susceptibility of the development stages of maize and the life cycle of *A. flavus*. These concerns were later addressed in a mechanistic model by Battilani et al. [17].

Model 3: AFLA-maize (Battilani et al., 2013): The AFLA-maize model [17] generates an index to predict the probability of exceeding the legal limit of AFB1. This prediction is based on a mechanistic approach, incorporating various stages of the infection cycle of *A. flavus*. It uses easily accessible weather variables (air temperature, relative humidity and rain) as inputs and was validated on Italian field data. The authors considered the inoculum source as non-limiting. The main components of this modelling approach were sporulation, infection, fungal growth and aflatoxin production at different temperatures and water activity levels. By completely abandoning the aridity index approach in their previous model, the authors ignored interactions happening due to the mismatch of soil moisture and its demand. This interaction, leading to development of drought and drought stress, seems to be a key factor in pre-harvest AF contamination. This model uses several sets of cardinal temperatures for different stages of the life cycle of *A. flavus*, like simulating growth or toxin production. However, these values and functions are based on in vitro trials which often are not appropriate to describe processes on the field. Other than drought stress (soil data and soil water processes), this model does not incorporate differences in susceptibility of hybrids and insect damage.

Model 4 (Chauhan et al., 2015): In a revised model, evaluated on maize grown in Kenya, Chauhan et al. [115] calculated an aflatoxin risk index based on water supply to demand ratios computed during the grain filling stages of maize. This model was also developed as a part of APSIM modelling framework where APSIM simulates maize growth, phenology and soil water balance using daily temperature, radiation and rainfall. In addition to daily weather variables, such a soil water balance model usually needs several other factors for describing the soil (e.g., field capacity, wilting point) and characterizing a plant (e.g., maximum rooting depth and plant height, length or temperature thresholds of different phenological stages). The authors used new cardinal temperatures on maize as a host plant for *A. flavus* growth based on their own in vitro trials. Unlike the model of Battilani et al. [17], the in vitro studies behind this model found that the optimal temperatures and temperature ranges for growth and AF production are similar. Inoculum, sporulation and germination in this model were considered non-limiting, water activity in grains before physiological maturity always being 1 (>24% grain moisture content) [46]. Trenk and Hartman [119] reported 17.5% moisture content to be a limit below which aflatoxin production will not occur. Considering drought stress as a driving factor for aflatoxin production, but using a mechanistic approach on the side of both the host plant and *A. flavus*, this model seems to mix and synthesize some major benefits of earlier models.

Model 5 (Damianidis et al., 2018): Damianidis et al. [117] calculated weekly drought indices (ARID) and found it useful to predict the risk for pre-harvest AF contamination in maize. Key factors for elevated risk of infection and AF level were drought stress around silking and during kernel development. The authors found a critical window between days 65 and 85 following planting when heat stress may result in increased AF contamination. Additionally, soil type and hybrid susceptibility to AF contamination proved to be statistically significant factors. Minimum temperature had the strongest positive influence on AF level over the 2 weeks after mid-silk. Low rainfall during the 2 weeks prior mid-silk and from day 43 after mid-silk to physiological maturity resulted in high AF contamination. According to these results, basic models incorporating minimum ambient temperature and the amount of precipitation could explain from 50 to 76% of the observed AF variability.

### 3.3. Opportunities and Limitations of Model Usage

An accurate prediction or estimation of the risk levels of AF contamination is of particular importance from the point of view of both healthcare and the economy. An easy to use mathematical model with good predicting capability is useful for growers, professional and specialist organizations (public health, farm advisory systems), competent authorities, economic operators (agricultural integrator companies, insurance and credit institutions) alike.

As we have seen based on the models introduced earlier, model developers try to aid this risk assessment work with different techniques. However, these approaches may present different challenges which users and decision makers should keep in mind. In addition, they should understand that a model is neither a panacea nor a perfect tool. While models have many uses in crop management, research and policy, they have their limitations.

Empirical, descriptive models are usually easier to comprehend, adapt and use, e.g., because of the fewer inputs needed, but they have to be calibrated to each new site and they cannot cope with a changing environment. Mechanistic models, on the other hand, are better able to model the interaction between the crop of interest and the environment. However, they can be more complex, making them more difficult to set up and use. They also may require more input data which may result in more accurate prediction, but not necessarily. If the required input data is unavailable and needs to be estimated or generated with a sub-model, the outputs may be even less stable compared to another model with fewer but reliable input data. Therefore, there are some cautions and limitations to consider by users and especially by developers of models.

Some simple, empirical, descriptive models use variables specific to the site and year the field trials have been carried out. Thus, these models have little predictive ability for growing seasons with unusual weather in a changing climate or for other locations. The extent of required input data is crucial. Some models use only meteorological variables and do not include effects specific to the field or management practices such as soil parameters, crop rotation, variety, and insect damage. Some models require variables not commonly used in agronomy or not assessed on a daily basis (e.g., a_w_), thus leading to estimations. Being aware of these limitations helps both the end user choosing the right model for the specific field of use and also the developer to construct a model which actually fits the reality regarding, e.g., the range of required input variables.

In addition to input data, another major barrier of widespread use may be the lack of comparability. Presently, model performance is hard to assess or compare because individual tools were usually validated on different, very limited sets of maize samples acquired from fields of a single geographical region. Another problem is that the depth of the publication of the models’ working mechanism is often not enough to reconstruct and validate them on an independent dataset.

Thus, assessment and comparison of models on an independent dataset from multiple geographical regions is required to help potential users and developers inciting widespread use and supporting focused development. Such a reference database should contain data from various regions and from many seasons. Beyond linked weather data and AF concentration of the sample, site specific soil data, plant specific (hybrid, phenological indicators) and agronomic factors (e.g., previous crop, tillage, dates of sowing and harvest, severity of insect damage) should be included in this database. Filtered datasets based on metadata can provide the basis for targeted testing and assessment of aflatoxin models aiming specific regions.

Additionally, online access to these models with integrated weather databases may increase the number of users, as it happened with irrigation models and services monitoring and predicting the occurrence of various pests.

For the success of aflatoxin models, however, improvement of the predicting capability seems to be a key factor. For this improvement, more field trials and continuous evaluation and validation based on field trial results is required.

## 4. Agronomy—Preventive and Corrective Steps

The presented models can indicate the likelihood of AF contamination, which makes them suitable for farmers to monitor their crops and make informed decisions about various corrective steps to be undertaken to minimize contamination. The corrective steps should generally aim at lowering stress to the plant caused by drought, excessive heat, inadequate plant nutrition, insect damage, weeds, excessive plant populations, and other plant diseases, which cause stress and facilitate fungal infestations [120,121,122,123,124,125,126,127,128,129,130,131,132,133,134,135,136,137,138,139,140,141,142,143,144,145,146,147,148,149,150,151].

Proper crop management and an early harvest may be efficient to reduce AF contamination in maize. During planting, the seeding rate can be varied between different risk zones, or more resistant corn hybrids can be planted in the high-risk areas. During crop growth, irrigation, pest and nutrient management strategies could be altered depending on the risk zones. Finally, at harvest, areas with different levels of risk can be harvested earlier or separately [88]. In years with higher AF risk indicated, an early harvest followed by rapid artificial drying [120] can be considered compared to leaving the crop in the field for dry down.

Three major trends to reduce pre-harvest *A. flavus* colonization and aflatoxin contamination can be identified in the literature: resistance breeding, biocontrol, and good agricultural practices (GAP). Genotype, biocontrol and most agricultural practices are used as prevention strategies, but there are some measures, which can be implemented later in the season when models indicate the risk of severe AF contamination.

### 4.1. Resistance Breeding

Resistance is important since *A. flavus* is a weak pathogen [121] and crops are frequently very resistant to infection and AF contamination unless environmental conditions favour fungal growth and crop susceptibility [122]. Corn is a good example of a crop containing heritable resistance to AF contamination [123,124]. Dynamics of water loss during maturity of maize ears appears to be determined by genotype, too. Breeding is, therefore, a possible strategy in controlling water activity of grain, which is a key parameter in modelling the colonisation and infection of *A. flavus* and other fungi [125].

As a fundamental step to precede successful resistance breeding of maize hybrids, Szabó et al. [126] urge the establishment of a screening methodology for ear infection severity and coverage. Production and identification of resistant hybrids and inbred lines is on the way because they can significantly reduce AF levels [127]. The SErAT program in the USA evaluated 295 hybrids and identified 13 of them with AF levels significantly lower than check averages [128]. Evaluations by Fountain et al. [129], Okoth et al. [130] in Kenya and South Africa, Brown et al. [124] with African maize lines in the USA, Guo et al. [131], Stagnati et al. [132] etc. resulted in several promising resistant lines and candidates for resistance breeding.

Since wounding by insects contributes to the severity of infections [122], some GMO hybrids with Bt traits may reduce AF levels. However, while hybrids with Bt traits had lower insect damage, in some years this may not be consistently correlated to AF levels [133] and Bt traits are not sufficient to reduce AFs [8].

### 4.2. Biocontrol

Biocontrol methods are one of the most widely researched innovations, especially the usage of atoxigenic *A. flavus* as a competitor for the aflatoxin producer one. Other methods include biopesticides/stimulants and other substances, such as essential oils extracted from herbs.

The application of atoxigenic isolates of *A. flavus* as biocontrol agents is a cost-effective and environmentally friendly way to reduce AF levels [134]. Carefully selected atoxigenic genotypes efficiently reduce AF contamination of crops when applied prior to flowering in the field. This method was pioneered in the US, but similar products with native strains (Aflasafe) have been developed for several African countries. Studies show that they reduce AF concentrations in treated crops by >80% in both field and storage conditions [135]. An aflatoxigenic *A. flavus* strain inside the kernels was suppressed by up to 82% when co-inoculated with a non-aflatoxigenic biocontrol strain. This resulted in a 73% suppression in AF levels [136]. An evaluation of atoxigenic strains in Italy resulted in an average AFB1 reduction of 92.3% with one strain [41].

Another trend in innovation is the elaboration of different application technologies. While higher or more consistent AF reduction may be possible via liquid delivery of biocontrol strains, the granular formulation of a non-toxigenic strain of *A. flavus* is also cost effective [137]. However, this method is not frequently adopted because of the challenge of applying a granular product through the canopy of maize in its reproductive stage. Weaver et al. [138] investigated a water-dispersible formulation of biocontrol strain in maize. When AF was present at very high levels, there was no treatment that could provide significant protection against AF contamination. The new formulation resulted in ≥49% reduction in all five treatments with a biocontrol strain.

Accinelly et al. [139] evaluated a leaf application method. The formulation was shown to be adherent, resulting in colonization of leaf surfaces with the biocontrol strain, and to reduce AF contamination of harvested kernels by up to 80% in Northern Italy and by up to 89% in the Mississippi Delta. The percentage of AF-producing isolates in the soil reservoir was not significantly changed by this application method.

There are promising biocontrol agents other than atoxigenic *A. flavus* strains. In South Africa, a *Trichoderma harzianum* strain was used to control *A. flavus* infection. In two field trials, AF levels of controls were 57 and 65% higher than those of the treated plants [140]. Treatment of crops with antagonistic strains of *Pseudomonas*, *Bacillus* and *Trichoderma* spp. resulted in lower infection levels [141]. Garcia et al. demonstrated that an extract of *Equisetum arvense* and a mixture of *Equisetum arvense* and *Stevia rebaudiana* is effective against growth of *A. flavus* and AF production [142]. More recently, Lagogianni and Tsitsigiannis evaluated six biopesticides/stimulants and found most of them effective in reducing conidia and/or AFB1 production in vitro, and two of them in situ [143]. Treatment with *Enterobacter cloacae* resulted in a reduced AF production by 96.9% [144].

Despite these promising results, biocontrol methods have their limitations. Interactions between different species and strains are in a state of flux and inhibitory effect varies under different environmental conditions. When water is freely available, atoxigenic and toxigenic strains may co-exist [145]. Additionally, proof of claimed efficacy for farms is difficult to obtain [146].

### 4.3. Good Agricultural Practice (GAP)

Good agricultural practice is the third researched group of measures and another approach to control *A. flavus* and AF contamination, especially by mitigating water stress, insect damage and generally lowering crop susceptibility. Organic agriculture systems appear to be similarly or less affected than conventional systems [147].

In agricultural practice, a possible tool to reduce *Aspergillus* infection is to apply chemical fungicides. Hayes et al. addressed the questions of tolerance to antifungal treatments and specifies the pathways of the antifungal effects of chemical fungicides [148].

It is noteworthy that manure facilitates growth of microorganisms that suppress soil infections [149]. Furthermore, a lower crop density may also attenuate fungal attacks on the crop. A plant density of 75,000 plants ha^−1^ (compared to 55,000 and 64,000 plants ha^−1^) increased fungal incidence [150], however, increasing the planting density from 44,480 to 74,130 plants ha^−1^ did not impact toxin accumulation in another study [151]. Attenuation via management of nutrition is complicated, but a higher proportion of maize plots had detectable AF contamination under low N supplementation in comparison to a higher N level in Kenya [152].

Planting date may also significantly affect the number of infected maize kernels. Delayed planting proved to reduce mycotoxin levels [151]. Maize test crosses showed doubly higher AF levels in the late-maturing group under low N treatment [152].

Milani [122] coupled delayed harvest and late irrigation during warm periods to increased AF levels. Nevertheless, any agricultural strategy that aims to reduce or eliminate drought stress may reduce AF contamination. However, prolonged irrigation caused increased kernel infection since ear rot incidence increased by 76.8%, compared to the control [153].

Parimi et al. have demonstrated the efficacy of education and raising farmer awareness: on-farm demonstrations of GAP resulted in a reduction of infections by 13–58% and AF levels of 62–94% compared to farmers’ practices [154].

## 5. Conclusions

The ongoing climate change may support higher yield levels and may also lead to a possible increase of AF contamination in maize production of the Pannonian agro-ecological zone. Nevertheless, literature addressing the predictable changes in this region and the feasible pre-harvest prevention measures is still limited.

Recognizing the health risks and economic impacts of AF contaminations, various models have been developed already to predict AF levels in different crops based on climatic data. In this paper, we introduced and compared several models with maize as a crop of interest.

To support assessment and comparison of models an independent dataset from multiple geographical regions is required. Such a reference database should contain data from various regions and from many seasons. Additionally, online access to these models and improvement of their predicting capability may increase the number of users. For this improvement, more field trials and continuous evaluation and validation is required.

While the introduced empirical, semi-mechanistic and mechanistic models may indicate the severity of *A. flavus* colonization and AF contamination later in the growing season, good agricultural practice seems to be a widely accepted general preventive measure against AF contamination, e.g., by reducing water stress and insect damage.

## Figures and Tables

**Table 1:** Minimum, optimum and maximum values of temperature (°C) and a_w_ (-) for growth and toxin production by *A. flavus* grown in different in-vitro mediums.

Substrate	Parameter	Growth			Aflatoxin Production		
		min	opt	max	min	opt	max
Whe at ^1^	Temperature	15	35	>42.5	15	25	42.5
	a_w_	0.80	0.95	>0.95	0.85	0.93	0.95
Sorghum ^1^	Temperature	15	37	ns	15	37	ns
	a_w_	<0.91	0.97	ns	0.94	0.97	ns
Rice ^1^	Temperature	20	33	42	<20	35	37
	a_w_	0.80	0.90	0.99	0.85	0.96	0.99
Malt extract-sucrose ^1^	Temperature	12–15	37	42	12	31	37
	a_w_	0.8–0.85	30–37	ns	0.85	0.99	0.99
Maize-based medium ^2^	Temperature	20	30	ns	ns	30	ns
	a_w_	0.90	0.99	ns	ns	0.98	ns
Other in vitro medium ^3^	Temperature	ns	35	ns	ns	33	ns
	a_w_	ns	0.95	ns	ns	0.99	ns

^1^ Adapted from [11], ^2^ [32], ^3^ [33], ns = not specified.

**Table 2:** Mathematical models to predict aflatoxin contamination in maize.

Year	Authors	Name	Characteristics
2003	Dowd, P. F. [116]	Mycotoxin predictor	Not published thoroughly.
2008	Chauhan et al. [83]	-	Semi-mechanistic daily model based on aridity index.
2008	Battilani et al. [10]	-	Empirical, decadal, aridity-based model.
2013	Battilani et al. [17]	AFLA-maize	Mechanistic model evaluated in Italy.
2015	Chauhan et al. [115]	-	Semi-mechanistic, aridity-based model evaluated in Kenya.
2018	Damianidis et al. [117]	-	Semi-mechanistic, weekly, aridity-based.
